# Transcriptome Approach Reveals the Response Mechanism of *Heimia myrtifolia* (Lythraceae, Myrtales) to Drought Stress

**DOI:** 10.3389/fpls.2022.877913

**Published:** 2022-07-08

**Authors:** Lin Lin, Jie Wang, Qun Wang, Mengcheng Ji, Sidan Hong, Linxue Shang, Guozhe Zhang, Yu Zhao, Qingqing Ma, Cuihua Gu

**Affiliations:** ^1^College of Landscape and Architecture, Zhejiang Agriculture & Forestry University, Hangzhou, China; ^2^Zhejiang Provincial Key Laboratory of Germplasm Innovation and Utilization for Garden Plants, Zhejiang Agriculture & Forestry University, Hangzhou, China; ^3^Key Laboratory of National Forestry and Grassland Administration on Germplasm Innovation and Utilization for Southern Garden Plants, Zhejiang Agriculture & Forestry University, Hangzhou, China

**Keywords:** RNA-Seq, ornamental plant, drought tolerance, plant hormone, transcription factor

## Abstract

Drought is a major environmental condition that inhibits the development and cultivation of *Heimia myrtifolia*. The molecular processes of drought resistance in *H. myrtifolia* remain unknown, which has limited its application. In our study, transcriptome analyzes were compared across three treatment groups (CK, T1, and T2), to investigate the molecular mechanism of drought resistance. Plant leaves wilted and drooped as the duration of drought stress increased. The relative water content of the leaves declined dramatically, and relative electrolyte leakage rose progressively. Using an RNA-Seq approach, a total of 62,015 unigenes with an average length of 1730 bp were found, with 86.61% of them annotated to seven databases, and 14,272 differentially expressed genes (DEGs) were identified in drought stress. GO and KEGG enrichment analyzes of the DEGs revealed significantly enriched KEGG pathways, including photosynthesis, photosynthetic antenna proteins, plant hormone signal transduction, glutathione metabolism, and ascorbate and aldarate metabolism. Abscisic acid signal transduction was the most prevalent in the plant hormone signal transduction pathway, and other plant hormone signal transductions were also involved in the drought stress response. The transcription factors (including MYB, NAC, WRKY, and bHLH) and related differential genes on significantly enriched pathways all played important roles in the drought process, such as photosynthesis-related genes and antioxidant enzyme genes. In conclusion, this study will provide several genetic resources for further investigation of the molecular processes that will be beneficial to *H. myrtifolia* cultivation and breeding.

## Introduction

Drought is a key abiotic stress factor affecting plant growth and development and threatening worldwide agricultural production, which restricts economic development and environmental governance (Fahad et al., 2017; Shah et al., 2017; Scharwies and Dinnen, 2019). Since the problem of serious global drought has increased with higher frequencies, longer durations, and wider ranges (Basu et al., 2016; Gupta et al., 2020), it has gradually become a common concern for an increasing number of plant species (Zhao X. et al., 2019; Qi et al., 2021). With the improvement in the quality of people’s life, the vision of building a garden city is strengthened, but urban population growth and industrialization have led to a prominent urban heat island effect, which exacerbates urban water shortages and high plant maintenance costs (Mccarthy et al., 2010; Ruosteenoja et al., 2018). Therefore, choosing drought-tolerant ornamental plants for a reasonable configuration would be very beneficial for water-saving garden construction (Zhang et al., 2019; Li et al., 2021). *Heimia myrtifolia* is a native tropical plant that grows beside streams and has good adaptability to high temperatures. After its introduction in China, it was used for garden applications and breeding germplasm. However, under seasonal drought with the absence of water, the growth of *H. myrtifolia* is significantly restricted and pollen vigor is reduced, which limits the progress of hybrid breeding and promotion. Therefore, it is necessary to explore the drought response mechanism of *H. myrtifolia* to provide a theoretical reference for subsequent breeding work and application.

Drought will cause a wide range of plant responses, such as decreasing cell osmotic potential, increasing reactive oxygen species (ROS), and cell membrane damage, inhibiting photosynthesis, and activating a large number of metabolic processes (Guo et al., 2018b; Kamanga et al., 2018; Dani and Siswoyo, 2019). During long-term natural selection and evolution, plants have formed a comprehensive regulatory mechanism for adaptation and resistance to drought stress, involving morphological, physiological, biochemical, and molecular mechanisms (Zhao D. et al., 2019). Plants will modify root architecture to absorb more moisture from the environment, and leaf stomata will be closed to reduce water evaporation (Buckley, 2019; Uga, 2021). Regarding physiological and biochemical processes, the enzymatic scavenging system can eliminate ROS caused by drought stress, as well as enzymes and reducing substances in the antioxidant defense system, including catalase (CAT), superoxide dismutase (SOD), and ascorbate peroxidase (APX) (Mittler et al., 2011; Fei et al., 2020). At the same time, photosynthesis is an important link for plants to synthesize organic compounds and conduct energy metabolism; they can adapt to arid environments by regulating stomata, enzyme activities, and photosynthetic pigments (Martin et al., 2017). When plants encounter drought stress, cells produce stimulatory responses, and the stress signals are transduced through transcription factors, protein kinases, and plant hormones. Then, a series of responses, such as osmotic regulation and photosynthesis, are activated (Wang et al., 2016; Cai et al., 2019). Signal transduction requires joint participation and regulation of a variety of hormones (Osakabe et al., 2014), and abscisic acid (ABA) is a well-studied plant hormone messenger molecule that is translocated from the sites of biosynthesis to guard cells; it further activates downstream signaling components, such as SnRK2s and MAPKs, which regulate stomatal closure (Kuromori et al., 2018). Most responses are normally controlled by transcriptional regulation involving transcription factors (TFs), which bind to the promoter regions of target genes and activate downstream gene responses. TFs closely related to drought stress include ABRE-binding factors (ABRE/ABF), WRKY, ABA-independent AP2/ERF, and NAC families (Takahashi et al., 2018; Yao et al., 2021). In conclusion, the response of plants to drought stress is an integrated process involving multiple signaling and gene expression changes that require comprehensive analysis.

With the development of sequencing technology and decreasing costs, transcriptomes play an important role in extensive areas, including abiotic stress resistance (Singh et al., 2017a). From the transcriptional level, it can be revealed that differential gene expression in plant signal transduction, endogenous hormones, photosynthesis, and other pathways under drought stress will deepen the comprehensive understanding of the mechanism of drought stress resistance (Sprenger et al., 2016). In recent years, an increasing number of studies have used sequencing technology to explore the molecular mechanisms of garden plants under drought stress, such as *Salix babylonica* and *Chrysanthemum rhombifolium*, and these results have greatly promoted drought resistance research on garden plants (Xu et al., 2021; Zhang et al., 2021). In research on *Pinus massoniana*, several transcription factor genes associated with the circadian rhythm (HY5 and LHY), signal transduction (ERF), and defense responses (WRKY) have been identified as playing a key role in adapting to drought stress (Du et al., 2018). Transcriptional regulation analyzes in peony (*Paeonia suffruticosa*) revealed that the synergy of the expression of *ERF* and *MYB* genes contributed to its resistance to varying degrees of drought stress (Guo et al., 2018a). Research has shown that rose (*Rosa chinensis*) transcription factors balance growth and drought survival, and the physiological and molecular mechanisms under drought stress have also been explored (Li et al., 2020b). Current drought resistance research mainly focuses on crops, while the research on ornamental plants is relatively rare, although it deserves in-depth attention and analysis.

*Heimia myrtifolia* (Lythraceae) is a deciduous shrub native to South America, ranging from Brazil to Uruguay, and is commonly known as “sun opener” or “shrubby yellow crest” (Rawat et al., 2007). It is of good ornamental value and breeding potential for its bright yellow petals and longstanding flowering period from August to September (Malone and Rother, 1994). *Heimia myrtifolia* can also be used as a medicinal plant with several pharmacologically active alkaloids and phenolics (Yang et al., 2000), which exhibit diuretic and strong anti-inflammatory activity (Lema et al., 1986; Ayoub et al., 2010). Despite its huge potential, *H. myrtifolia* is mainly restricted by seasonal drought and less moisture against its origin. Based on previous research, the physiological regulation mechanism of *H. myrtifolia* to resist drought stress has been revealed, but the molecular mechanisms that account for the adaptation of *H. myrtifolia* to drought remain unclear, limiting its introduction and application. Herein, we used RNA-Seq to explore the molecular mechanism and identify the metabolic pathways and genes enabling resistance to drought to comprehensively explain the drought resistance mechanism. This research will provide extensive genetic resources for further molecular biology research, drought-resistant cultivation, and breeding.

## Materials and Methods

### Plant Material, Treatments, and Physiological Analysis

Uniformly sized 1-year-old cut seedlings of *H. myrtifolia* were obtained from the Zhejiang A&F University intelligent greenhouse, Hangzhou, Zhejiang province, China (30°13′48″N, 119°43′12″E). Each pot contained a single plant and was filled with 4 kg of soil. The soil water content was maintained at field capacity (33.5%). Before the water-deficit treatments, the seedlings were incubated in an artificial climate room with 70% humidity and light (14 h, 28°C)/dark (10 h, 25°C). Three days before the drought stress treatment, all materials were watered thoroughly every day. The natural drought method without watering was adopted using a TDR100 portable soil moisture meter (Spectrum, Aurora, IL, United States) to measure the soil moisture content. The seedling pots were weighed and watered at 8:00 a.m. every day to maintain each treatment within the control range. The drought treatment continued for 12 days. The relative soil water content of the control treatment (CK) was 65–75%, and the relative soil water content of the two stress treatments was maintained at 30–45% (T1) and 15–25% (T2). The phenotype, relative water content (RWC), and relative electrolyte leakage (REL) of the leaves (mature leaves at the same height and the same orientation in the upper part) were determined using the method presented by Galmés et al. (2007) and Sun et al. (2020). The leaves were collected for physiological experiments and transcriptome sequencing, and three biological replicates for each sampling time were immediately collected and stored at –80°C.

### RNA Extraction, cDNA Library Construction, and Transcriptome Sequencing

Total RNA was extracted from the leaves using TRIzol reagent (TaKaRa, Inc., Dalian, China) according to the manufacturer’s instructions (Zhang et al., 2016). The total RNA was quantified using NanoDrop and an Agilent 2100 bioanalyzer (Thermo Fisher Scientific, Waltham, IL, United States). To prepare sequencing libraries, oligo(dT)-attached magnetic beads were used to purify mRNA. Fragmentation buffer was added to break the mRNA into short fragments. mRNA was used as a template, and random hexamers were used to synthesize the first-strand cDNA. Then, buffer, dNTPs, RNase H, and DNA polymerase I were added to synthesize the second-strand cDNA. The cDNA fragments obtained from the previous step were amplified by PCR, and products were purified by AMPure XP Beads and then dissolved in EB solution. The double-stranded PCR products from the previous step were heated denatured and circularized by the splint oligo sequence to obtain the final library. Single-stranded DNA was used as the format for the final library.

### *De novo* Assembly, Functional Annotation, and Classification of Unigenes

We used Trinity software (v2.8.0^1^) with default settings for *de novo* transcriptome assembly. The two obtained contigs were connected into a single scaffold to generate unigenes (Grabherr et al., 2011). These unigenes were further spliced to generate longer complete consensus sequences, and redundant sequences were removed with Tgicl (v2.1^2^) (Pertea et al., 2003). The unigene sequences were aligned to seven functional databases (KEGG, GO, NR, NT, Swiss-Prot, Pfam, and KOG), employing BLAST (v2.2.23) with system default parameters and a threshold of e ≤ e^–10^ (Zhou et al., 2016). Based on the NR annotation, GO functional annotation was obtained using Blast2GO software (Conesa and Götz, 2008). The pathway assignments were conducted by performing sequence searches against the KEGG database using the BLASTX algorithm with an E value threshold of 10^–5^ (Mcginnis and Madden, 2004).

### Analysis of Differentially Expressed Genes (DEGs)

Bowtie2 was used to align clean reads to the reference sequences that were composed of all obtained transcript sequences, and RSEM was used to calculate the gene expression level of each sample (Li and Dewey, 2011; Langmead and Salzberg, 2012). The relative gene expression level between different samples was calculated using the log2 ratio. Fragments per kilobase of exon model per million mapped fragments (FPKM) of each gene were calculated based on the length of the gene and reads count mapped to this gene. Differential expression between the two conditions was analyzed using the DESeq2 method (Love et al., 2014). Genes, with | log2FC| > 1 and a *p*-value < 0.05, were considered as DEGs (Anders and Huber, 2010). The DEGs were analyzed for GO functional classification and KEGG pathway enrichment analysis, and *P-*value ≤ 0.05 was the threshold of significance for the GO terms and KEGG pathways (Minoru et al., 2004).

### The qRT-PCR Validation for Differentially Expressed Genes (DEGs)

According to the gene annotation results, nine DEGs were selected for qRT-RCR validation experiments. These genes were randomly selected among the DEGs that were closely related to drought stress and had large differential expression fold changes. The primers were designed using Primer Premier 5.0 software (Supplementary Table 1). cDNA was transcribed from 5 μg of total RNA using the PrimeScript™ First-Strand cDNA Synthesis Kit in 20 μL of the reaction mixture. RT-qPCR was performed in an ABI7500 Real-Time System (Applied Biosystems) using SYBR Green I (Roche). The amplification procedures were as follows: 95°C for 10 min, followed by 40 cycles of 95°C for 15 s, 58°C for 10 s, and 72°C for 25 s. Three biological and technical replications were performed for each sample. The relative expression levels were normalized to the expression of the *GAPDH* gene (Chen et al., 2021). Data are presented as relative transcript levels based on the 2^–ΔΔ*Ct*^ method (Livak and Schmittgen, 2001).

### Statistical Analysis

Data were analyzed with the R software^3^ using the one-way analysis of variance (ANOVA) for significant difference. The error bars were calculated with data from three replicates. ANOVA results were considered significant at *p* < 0.05, and mean comparisons were made using the Tukey HSD test.

## Results

### Phenotypic and Physiological Responses

**FIGURE 1:**
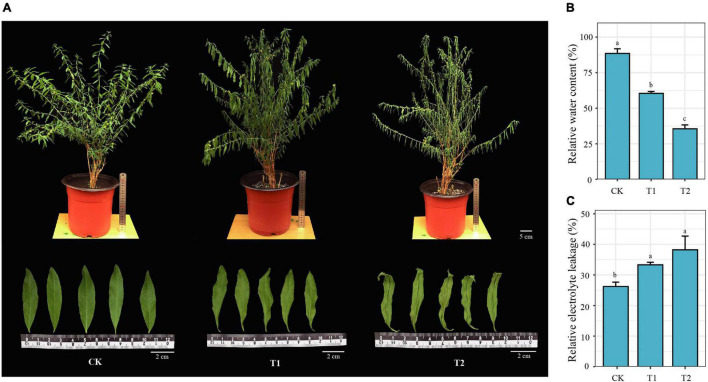
Phenotypic and physiological responses of *Heimia myrtifolia*. **(A)** Morphological observation of *H. myrtifolia* in response to different drought stress treatments. **(B)** Relative water content (%). **(C)** Relative electrolyte leakage (%). Different letters indicate the result is significantly different (*p* < 0.05).

As the primary organ for photosynthesis, leaves can effectively reflect a plant’s drought adaptability. Three treatment groups of *H. myrtifolia* leaves were photographed and collected (CK, T1, and T2). The healthy CK leaves were lanceolate, and the leaf color was bright green. The edges of the leaves were slightly curled, but the branches were significantly drooping in T1. The leaves were severely curled, and chlorosis of the leaves was obvious at T2 (Figure 1A). Plant morphology was also affected; growth and development were inhibited, and the leaves withered and fell off. The RWC gradually decreased from 92.18% in CK to 60.45% (T1) and 35.54% (T2) in the treatments (Figure 1B). Drought stress can damage plant cell membranes, cause changes or loss of plant cell membrane permeability, and cause electrolyte extravasation (Susilo et al., 2019). In this experiment, with drought increases, the REL of the leaves showed an increasing trend, reaching a peak at 38.23% in T2 (Figure 1C).

### Overall Summary of Transcriptome Data

The sample sequencing quality evaluation results showed that nine samples obtained 43.02–49.08 M raw reads (Table 1). Raw reads removed linker sequences, duplicates, and low-quality fragments. A total of 37.63–43.46 M high-quality clean reads were obtained, and the GC content was 46.81% on average. After filtering out the unqualified reads in the original data, the effective data volume was 56.44 Gb, and the effective rate was between 86.89 and 89.41%. The proportion of data with data quality ≥ Q20 was above 93.77%.

**TABLE 1:** Summary of RNA-sequencing data under drought stress.

Sample	Total raw reads (M)	Total clean reads (M)	Total clean bases (Gb)	Clean reads ratio (%)	Q20 (%)	GC (%)
CK_1	49.08	42.94	6.44	87.50	95.65	46.80
CK _2	49.08	42.68	6.40	86.96	95.52	47.00
CK _3	47.33	42.31	6.35	89.41	95.28	47.27
T1_1	49.08	43.46	6.52	88.55	95.41	46.73
T1_2	43.02	37.63	5.65	87.48	94.1	47.29
T1_3	48.95	42.72	6.41	87.28	93.77	46.69
T2_1	49.08	42.65	6.40	86.89	93.78	46.55
T2_2	44.53	38.81	5.82	87.15	93.88	46.69
T2_3	49.08	43.03	6.45	87.67	93.77	46.30

*≥ Q20 (%) indicates the percentage of bases with the quality value ≥ 20 in clean data.*

Using Trinity software to *de novo* assemble the high-quality data of nine samples, 62,015 unigenes were obtained. The total number of unigenes was 107,289,516 bp with an average length of 1730 bp. The N50 length was 2510 bp, and the N90 length was 948 bp (Supplementary Figure 1). To fully understand gene function, the transcriptome sequences were compared to seven functional databases (Supplementary Table 2), and 62,105 unigenes were annotated. The number of unigenes compared with the NR database was the largest, with 51,982 (83.82%) unigenes. The number of unigenes successfully annotated to the NT, Swiss-Prot, KEGG, KOG, Pfam, and GO databases was 45,155 (72.81%), 40,806 (65.80%), 42,435 (68.43%), 42,089 (67.87%), and 39,212 (63.23%), respectively. Among them, there were 23,739 (38.28%) unigenes that could be successfully annotated in the seven major databases, and the number of genes annotated in at least one database was 53, 433 (86.16%).

**FIGURE 2:**
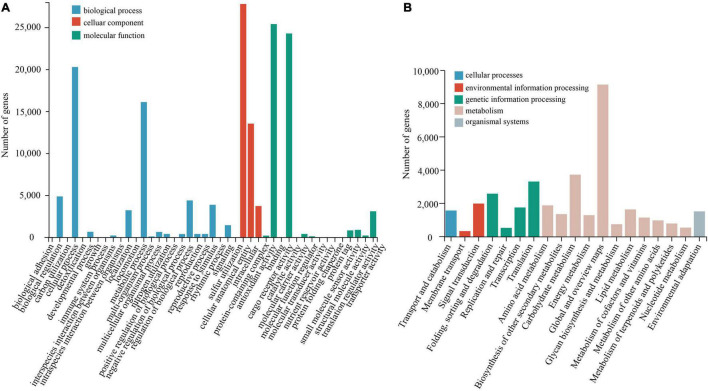
Functional and pathway enrichment of unigenes. **(A)** GO enrichment with three primary classifications of biological process, cellular component, and molecular function. **(B)** Functional classification and pathway assembled unigenes by KEGG.

Compared with the NR database, the species with the greatest homologous sequence alignments was *Punica granatum* of the Lythraceae family, with 43,143 (83%) homologous sequences (Supplementary Figure 2). The KEGG annotation results (Figure 2A) showed that the most represented metabolic pathway was carbohydrate metabolism (3764, 10.03%). The main basic metabolic pathways included amino acid metabolism (1916, 5.10%), lipid metabolism (1675, 4.46%), and transportation and catabolism (1608, 4.28%). Using the KOG database to classify the proteins (Supplementary Figure 3), 25 KOG functional categories were annotated. Among these functional categories, general function predictions only category when compared with 8761 genes (20.65%), accounted for the largest proportion, showed that some new proteins with unknown functions were found. This was followed by signal transduction (5752, 13.5%) and translation modification (3627, 8.55%). The analysis of GO enrichment was performed using Blast2GO software (Figure 2B), and GO functions were mainly enriched in three aspects: biological process, cellular component, and molecular function (Harris et al., 2004). The distribution of GO functions involved 43 functional subcategories, and the biological process contained 25 functional subcategories, of which the most functional subcategory was cellular anatomical entity and binding, with 27,870 (17.41%) and 24,356 (15.22%) unigenes, respectively. GO subclass functions were related to the drought stress response, which are mainly related to antioxidant activity, transcription regulation, and signal transmission.

### Expression and Functional Analysis of Differentially Expressed Genes (DEGs)

**FIGURE 3:**
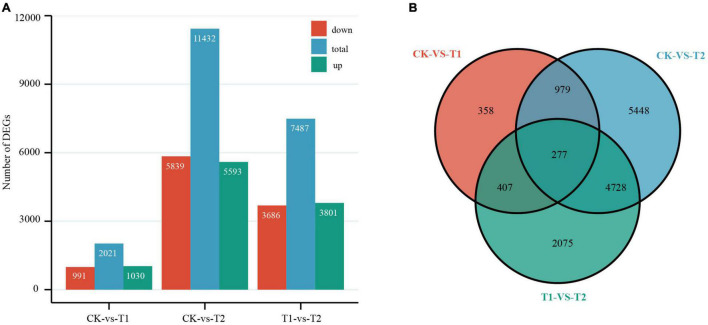
Differentially expressed genes (DEGs) in *Heimia myrtifolia* during drought stress. **(A)** The number of DEGs between CK and drought treatment group in T1 and T2. **(B)** Venn diagram representing DEGs expressed after drought treatment.

By comparing the number of independent fragments in the three libraries to reflect the relative abundance of mRNA, using the control treatment (CK) as the reference value, pairwise comparisons were made between the two treatment groups (T1 and T2) and CK. The threshold of DEGs was | log2 fold change| ≥ 1 and *P*-value ≤ 0.05. DEGs that met the criterion log2 fold change > 0 were considered upregulated, and others were considered downregulated (Anders and Huber, 2010). From the Venn diagram and histogram (Figure 3), a total of 14,272 DEGs were detected, of which 277 genes were differentially expressed among the two treatment groups, and 358 and 5448 DEGs were specifically expressed in T1 and T2, respectively. There were 2021 DEGs (1030 were upregulated and 991 were downregulated) between CK and T1, and a total of 11,432 DEGs (5593 were upregulated and 5839 were downregulated) were detected between CK and T2. Furthermore, principal component analysis showed good intergroup correlation and high repeatability (Supplementary Figure 4); therefore, the samples were reliable for use in subsequent data analysis.

GO enrichment analysis (Supplementary Figure 5) showed that DEGs were enriched in 41 GO terms (Supplementary Table 3), and the least GO terms enriched to cell components, which was consistent with the result that unigenes were successfully annotated into the GO database. “Cellular anatomical entities” were enriched with the largest number of genes, with 787 and 4592 genes enriched in T1 and T2, respectively. “Catalytic activity” (733 DEGs in T1, 4173 DEGs in T2) and “binding” (727 DEGs in T1, 4197 DEGs in T2) in the “molecular function” category were significantly enriched. In the “biological process” category, “cellular process” and “metabolic process” were significantly enriched. The top GO terms enriched at the two time points were roughly similar, but the number of differential genes enriched in T2 increased significantly. The GO terms related to drought stress were “antioxidant activity,” “cell redox homeostasis,” “photosynthesis,” “response to heat,” and “signaling.” DEGs involved in these terms were worthy of follow-up attention.

**FIGURE 4:**
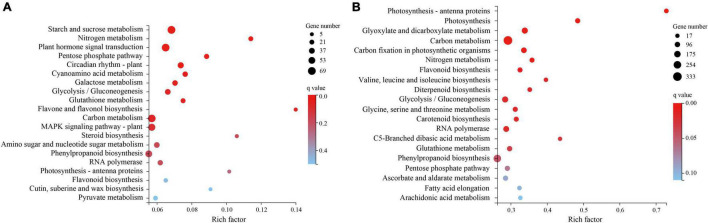
KEGG pathway enrichment with differentially expressed genes (DEGs) between different droughts treated. **(A)** KEGG pathway enrichment of DEGs to drought stress in T1. **(B)** KEGG pathway enrichment of DEGs to drought stress in T2.

The KEGG pathway enrichment analysis (Figure 4) found that the plant hormone signal transduction pathway (KO: 04075) was significantly enriched in T1 (Supplementary Table 4). Three pathways closely related to drought resistance were significantly enriched in T2 (Figure 4B): photosynthesis–antenna proteins (KO: 00196), photosynthesis (KO: 00195), and glutathione metabolism (KO: 00480). Amino acid metabolism, biosynthesis of other secondary metabolites, carbohydrate metabolism, and lipid metabolism were the top four enriched pathways with 400, 219, 104, and 68 DEGs enriched in T1, respectively, and 2223, 946, 683, and 401 DEGs enriched in T2, respectively. The DEGs enriched in these four metabolic pathways may be involved in *H. myrtifolia*’s drought response. The top 20 KEGG pathway results revealed that plant hormone signal transduction pathways, photosynthesis pathways, photosynthesis–antenna proteins pathways, glutathione metabolism pathways, and ascorbate and aldarate metabolism pathways were significantly enriched, which played an important role in the process of drought stress and mainly regulated the expression of downstream functional genes in response.

### Regulation of the Plant Hormone Signal Transduction Pathway to Drought Stress

**FIGURE 5:**
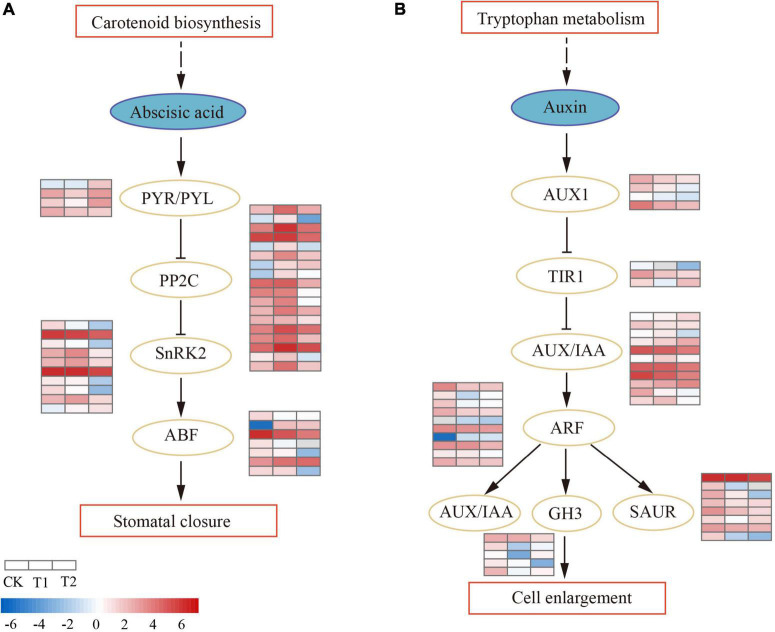
Differential regulation of plant hormone signal transduction pathway in drought treatments. The heatmaps show log_2_FPKM values of the differentially expressed genes (DEGs). **(A)** Abscisic acid signal transduction pathway. **(B)** Auxin signal transduction pathway.

The plant hormone signal transduction pathway (ko04075) was significantly enriched (*P* < 0.05) in T1, and most DEGs were involved in the abscisic acid (ABA) and auxin (AUX) pathways (Supplementary Table 5). In the ABA signal transduction pathway (Figure 5A), ABA receptor PYR/PYL was downregulated in T1, while two (*CL2679.Contig1_All* and *CL2679.Contig2_All*) were upregulated and one (*Unigene417_All*) was downregulated in T2. The activity of PYR/PYL enhanced the downstream negative regulatory protein factor PP2C. Eighteen DEGs were annotated as PP2C; PP2Cs were all upregulated in T1, most were downregulated in T2, and only one PP2C (*CL6736.Contig5_All*) was continuously upregulated in both treatments. Protein kinase SnRK2 was peculiarly expressed in T2, except for one SnRK2 (*CL1848.Contig8_All*) that was upregulated in T2; nine SnRK2s were downregulated. The downstream ABA-responsive transcription factor ABF decreased activity and inhibited the expression of ABA-responsive genes. In auxin hormonal signaling (Figure 5B), there were 11 *AUX/IAA* genes. Only one (*CL7309.Contig2_All*) was specifically upregulated in T1, most were downregulated in T2 (three upregulated, seven downregulated), and one *AUX/IAA* gene (*Unigene9101_All*) was downregulated in both treatments. A total of 10 differentially expressed auxin regulator ARFs (two upregulated and eight downregulated) were identified. ARF downregulated downstream early auxin response genes *SAUR*, *TIR1*, and *GH3* in the drought process. The brassinosteroid biosynthesis pathway was enriched (*P* < 0.05) in T2. In brassinosteroid (BR) signaling, the membrane receptors BRI1 and BAK1 had enhanced activity, both of which were upregulated in T1. BSK (BR-signaling kinase) was mainly upregulated in T2, which eventually led to the downregulation of *TCH4* genes and D-type cyclin (CYCD3) expression (Supplementary Table 6).

### Regulation of Photosynthesis to Drought Stress

The photosynthesis–antenna protein pathway (ko00196) was significantly enriched in both T1 and T2. Many photosystem II light-harvesting chlorophyll a/b-binding proteins, such as *Lhcb* genes, were differentially expressed, and most of them were activated at T2 (Supplementary Table 7A). There were two and 37 *Lhcb* genes downregulated in T1 and T2, respectively. Fifty-nine DEGs were enriched in the photosynthesis pathway (ko00195) in T2. These DEGs were mainly composed of the photosystem I protein *Psa* gene, photosystem II protein *Psb* gene, photosystem I reaction center protein gene, photosystem I chlorophyll apolipoprotein gene, and photosystem II protein gene. A total of four *Psa* genes and eight *Psb* genes were found, and the FPKM values of some photosynthesis-related DEGs were up to 1000 or higher (Supplementary Table 7B). The pathways of porphyrin and chlorophyll metabolism (ko00860) were also significantly enriched in T2, and 45 DEGs were enriched. Chlorophyll was catalyzed by a series of enzymatic reactions, and the final step was chlorophyll synthase (CHLG), which catalyzed the synthesis of chlorophyll a/b. In our study, two chlorophyll degradation key enzymes, *CAO* (*CL2647.Contig2_All* and *CL2647.Contig3_All*), were downregulated in T2, and one *CHLG* gene (*Unigene5893_All*) was also differentially downregulated in T2 (Supplementary Table 7C).

### Regulation of Active Oxygen-Scavenging System to Drought Stress

In our study, glutathione metabolism (ko00480) and ascorbate and aldarate metabolism pathways (ko00053) were significantly enriched in T2, which were enriched 87 and 89 DEGs, respectively. A total of 21 glutathione-S-transferase (GST) DEGs were identified (Supplementary Table 8A). Five *GST* genes were upregulated and seven were downregulated in T1, while in T2, most *GST* genes were upregulated. Two differentially expressed glutathione peroxidase (GSH) genes were also identified during drought stress, and both *GSH* genes were upregulated in T2. Five DEGs of dehydroascorbate reductase (DHAR) were identified in the ascorbate and aldarate metabolism pathways. The expression of five *DHAR* genes was significantly changed in T2 (three upregulated and two downregulated), one (*Unigene10653_All*) of which was upregulated in both drought periods. Four of the six ascorbate peroxidase (APX) genes were downregulated in T2, and *APX* genes that were upregulated in T1 had a higher expression than that observed in T2 (Supplementary Table 8B). These two pathways were significantly enriched, suggesting that related antioxidants play an important role in eliminating oxidative damage caused by drought stress. In our study, 26 differentially expressed antioxidant enzyme genes were also identified, including 14 *POD* genes, 10 *CAT* genes, and two *SOD* genes (Supplementary Table 8C). Both *SOD* genes were upregulated in T1. The *CAT* genes were mostly downregulated in T2, and two were upregulated (*CL654.Contig3_All* and *CL654.Contig14_All*) in both periods. Fourteen *POD* genes (among them four upregulated in T1 and one downregulated in T1) were identified. The *POD* genes mainly changed in T2, seven were upregulated, and five were downregulated.

### Major Transcription Factors Differentially Regulated Under Drought Conditions

**FIGURE 6:**
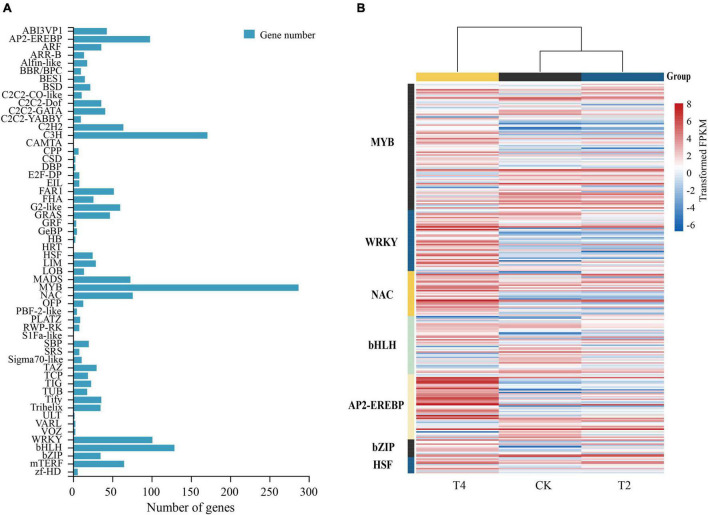
Differential regulation of transcription factors in drought treatments. **(A)** The number of differentially expressed genes (DEGs) of transcription factors after drought treatment. **(B)** Heatmap of seven classes of transcription factors (MYB, WRKY, NAC, bHLH, AP2-EREBP, bZIP, and HSF) closely associated with drought; the heatmap shows log_2_FPKM values of the differentially expressed TFs.

Using the PlantTFDB plant transcription factor database, 1901 encoding transcription factors (TFs) belonging to 56 gene families were identified in *H. myrtifolia*’s transcriptome database (Figure 6A). The top five TF families were MYB (287, 15.10%), C3H (171, 9.01%), bHLH (129, 6.79%), WRKY (101, 5.31%), and AP2-EREBP (98, 5.16%). Differentially expressed TFs were displayed in an expression heatmap (Figure 6B), including the common TF families MYB, bHLH, AP2-EREBP, NAC, and C2H2. In our study, the DEGs of the MYB family accounted for the largest proportion. A total of 93 MYB TFs were differentially expressed under drought stress, with 25 MYB TFs (10 upregulated and 15 downregulated) expressed in T1. A large number of MYB TFs (42 upregulated and 43 upregulated) were specifically expressed in T2, but the downregulated MYB TFs were generally higher in expression. Most of the NAC TFs were upregulated, with nine being upregulated in T1. A total of 27 NAC TFs were upregulated in T2, and six were upregulated in both treatments. Similarly, WRKY TFs were upregulated during drought stress, and 39 were differentially expressed in T2, of which 35 were significantly upregulated. Only one WRKY TF was upregulated, but eight WRKY TFs were downregulated in T1. Ten bHLH TFs were upregulated, and 16 were downregulated in T1. A total of 12 bHLH TFs were upregulated and 22 were downregulated in T2, of which five were upregulated in both treatments. Most of the AP2-EREBP TFs were upregulated, five were upregulated, and five were downregulated in T1. In T2, 15 were downregulated and 35 were upregulated. The upregulated AP2-EREBP TFs maintained a high expression level. Thirteen differentially expressed bZIP TFs were identified, of which three and nine were upregulated in T1 and T2, respectively. HSPs changed significantly and played an important role under drought stress, while the heat shock transcription factor (HSF) regulated the expression of HSPs. Twelve differentially expressed HSF TFs were found in both treatments and were mostly upregulated.

### Real-Time qPCR Validation

**FIGURE 7:**
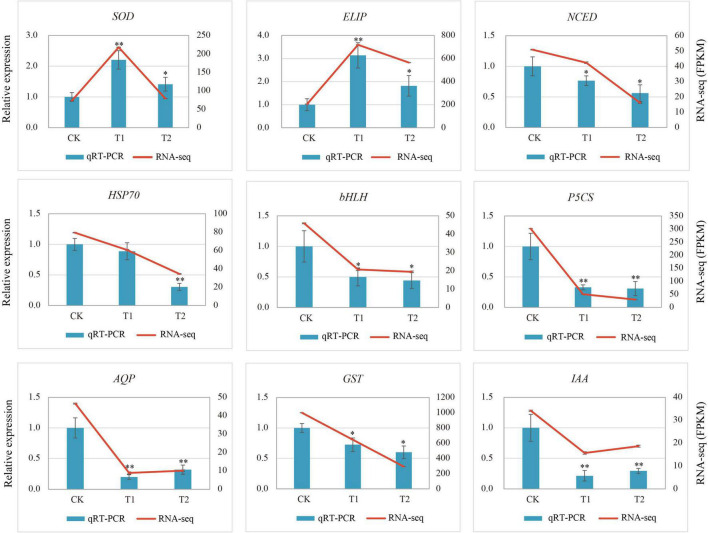
Relative gene expression of nine differentially expressed genes (DEGs) in response to drought stress. Vertical bar charts with error bars (left y-axis) represent the quantification results of nine DEGs using RT-qPCR, and the values are means ± SE (*n* = 3). Line graphs (right y-axis) represent the transcript abundance (FPKM) of each gene detected by RNA-Seq. Asterisks (* or ^**^) represent the significant differences at *p* < 0.05 or *p* < 0.01 when compared with the control, respectively.

To verify the reliability of the transcriptome results, nine DEGs related to drought stress were selected and the specific primers were designed for qRT-PCR. The relative expression of the genes in the qRT-PCR analysis results and the FPKM value in RNA-Seq were used for the validation of sequencing data. The qRT-PCR results of nine genes (Figure 7) were slightly different from the expression in the sequencing data, but the overall expression trend was basically the same. The correlation of the linear regression analysis was 0.8132, which further verified the accuracy and credibility of the transcriptome data (Supplementary Figure 6).

## Discussion

### Key Pathways and Drought Tolerance-Related Genes Under Drought Stress

Among the KEGG pathways enriched under drought stress, the photosynthesis–antenna protein pathway was significantly enriched in both periods, and the photosynthesis pathway and porphyrin and chlorophyll metabolism pathway were also significantly enriched in T2, indicating that the regulation of the photosynthetic system plays an important role under drought stress. Plant photosynthesis is a complex physiological process and an important pathway for plant energy synthesis and metabolism, and drought stress affects stomatal opening and the CO_2_ transfer rate, limiting plant photosynthesis (Banks, 2018). Photosystem II is a critical mechanism for photosynthesis and energy conversion in plant chloroplasts, converting electrical energy into active chemical energy (Hu et al., 2018). In our study, the photosystem I and II protein genes and light-harvesting pigment protein genes were downregulated during drought stress. The photosystem II light-harvesting chlorophyll a/b-binding protein *Lhcb* gene was significantly downregulated in T2, which maintained a high expression level before downregulation. The results indicated that the light-responsive process was disrupted to a certain extent, and the roles of *Lhcb* genes in regulating drought-induced responses are relatively active. Similar to the results of our study, the downregulation of DEGs related to chlorophyll degradation and photosynthetic capacity in *Paeonia lactiflora* severely damaged the photosynthetic system and reduced the ornamental value (Li et al., 2020a). In our study, the photosynthetic protein genes *Psa* and *Psb* were also downregulated under drought stress, and the D1 protein in the light reaction center was damaged. Therefore, the photosynthetic system may be inhibited by non-stomatal factors. Photosynthetic pigments are a significant medium for plants to convert inorganic substances into organic substances, and the excitation of chlorophyll molecules by light will transfer electrons and synthesize ATP (Lang et al., 2018; Wang et al., 2018). In our study, two chlorophyll b synthesis *CAO* genes and one chlorophyll synthesis *CHLG* gene were downregulated in T2. The results may indicate that the chlorophyll synthesis process was hindered to some extent, and the leaf color gradually became lighter during the stress process, from tender green to yellow–green. In this study, the photoreaction system of *H. myrtifolia* was destroyed under drought stress, and the expression of many photosynthesis-related genes was downregulated, resulting in a decrease in the photosynthetic rate and blocking chlorophyll synthesis. However, the energy absorbed by photosynthesis still plays a protective role and maintains metabolism.

Drought stress increased the production rate of reactive oxygen species (ROS), and excessive ROS induced oxidative damage in plant membrane systems; thus, the balance of ROS was closely related to peroxidase pathways (Zhang et al., 2019). In our study, the relative conductivity (REL) of *H. myrtifolia* gradually increased with drought stress, indicating that the membrane system was damaged by oxidation. The ascorbic acid–glutathione system is an important mechanism of the antioxidant system in plants, and these two pathways were also significantly enriched in our study (Aguiar et al., 2016). Glutathione-S-transferase in the glutathione pathway is a multifunctional enzyme that can degrade harmful substances and reduce cell damage. The *APX* genes and *GST* genes help remove excess reactive oxygen species (Dalal et al., 2018; Rehman et al., 2021). In our study, 17 *GST* genes were identified, most of which had high expression levels, which were consistent with a previous study on *Masson pine*, with an upregulated expression of many genes in the antioxidant defense system (Haas et al., 2021). The results showed that the glutathione metabolic pathway played an active and synergistic role in scavenging excess reactive oxygen species. DHAR is a plant-specific glutathione-S-transferase that catalyzes the reduction in dehydroascorbic acid to ascorbic acid (Li et al., 2018). In our study, five differentially expressed *DHAR* genes were identified and were shown to assist plants in producing ascorbic acid to enhance their antioxidant capacity. SOD, POD, and CAT enzymes are also important antioxidant enzymes for free radical scavenging in plants, and the genes regulating these enzymes were also differentially expressed in our study (Rahimi et al., 2021). Two *SOD* genes in our study were upregulated in T2, but most of the 10 *CAT* genes were downregulated in T2. *POD* genes were also mainly differentially expressed in T2, indicating that the generation and clarity of hydrogen peroxide during the stress process were basically the same. The antioxidant enzyme genes actively participate in the stress resistance response and jointly remove superoxide-free radicals and H_2_O_2_ in plants, alleviating the damage caused by drought stress.

### Drought Stress Regulates the Plant Hormone Signal Transduction Pathway

Plant hormones are key regulators of plant growth and development. Plant hormones can mediate signal transduction, which is directly involved in regulating the drought response process (Gupta et al., 2020; Sun et al., 2020). The plant hormone signal transduction pathway was significantly enriched (*P* < 0.05) in T2. ABA is an important plant hormone in the abiotic stress response, and drought stress can induce the production of ABA in the root system. ABA signal transduction to the leaves causes the stomata to close, which inhibits photosynthesis in plants (Kuromori et al., 2018). A study found that ABA signaling-related genes were susceptible to drought stress in *Pinus massoniana*, such as PYL and PP2C, which regulates the expression of stress-responsive genes (Péret et al., 2012). ABA signaling is needed to recognize ABA and initiate to receive the subsequent signal transduction process. The ABA receptor PYR/PYR protein was located the most upstream of the ABA signal transduction pathway, and the transcription efficiency of PYL significantly affected the rate of ABA signal transduction (Peleg and Blumwald, 2011; Takahashi et al., 2018). PYR/PYL and PP2C, as part of the ABA signaling pathway, were upregulated in our study. Between the two treatments, four *PYR/PYL* genes were differentially expressed. The PYR/PYL genes formed a complex with PP2Cs, and the expression of PP2Cs was negatively regulated, which was consistent with the results from previous studies on *Oryza sativa* (Ramachandran et al., 2021). The decreased activity of PP2C led to the downregulation of the protein kinase SnRK2, which ultimately led to increased ABA signaling intensity, activation of stomatal closure, and restricted root growth. The results suggest that ABA signaling plays an active role in improving drought resistance in *H. myrtifolia*. Auxin signal transduction, which can regulate root growth, leaf development, and phototropic growth, among other processes, also played an important role in our study (Liang et al., 2019). The auxin-responsive genes *AUX/IAA* in our study were mainly downregulated in T2. The auxin regulatory factor (ARF) and the auxin early-responsive genes *SAUR* and *GH3* were mainly downregulated under drought stress. Similar to the results obtained in *Benincasa hispida*, *SAUR* genes were less expressed in drought-tolerant cultivars, reflecting better regulation of penetration (Wang et al., 2019). ARF TFs can regulate hormonal signaling to maintain normal plant growth under stress (Péret et al., 2012). AtARF2 in *Arabidopsis thaliana* is involved in the regulation of ethylene and auxin signaling pathways (Singh et al., 2017b). In this study, the downregulated expression of *SAUR*, AUX/*IAA*, and *GH3* genes may be due to the inhibition of auxin synthesis during drought stress resistance, which improved the drought resistance of *H. myrtifolia* and slowed the growth of plants to adapt to the external environment. In BR signaling, the expression of membrane receptor proteins BRI1 and BAK1 was activated, and the activity of D-type cyclin was inhibited, which affected plant cell division (Ye et al., 2010). The enhanced activity of membrane receptors BRI1 and BAK1 in this study positively regulated plant adaptation to drought. In this study, plant hormones actively transmitted stress signal molecules, regulated downstream gene expression with transcription factors, and participated extensively in the adaptation of *H. myrtifolia* to the environment.

### Role of Transcription Factors in the Drought Stress of *Heimia myrtifolia*

In our study, 1901 encoding TFs belonging to 56 gene families were identified. MYB, WRKY, and bHLH TFs are among the top five gene families that have been identified to be closely related to hormone regulation and play an important role in the drought tolerance process of plants (Wang et al., 2016). MYB is an important TF involved in cell differentiation and root growth and induces the expression of ABA-related genes under drought stress (Imran et al., 2018). Overexpressing MYB2 transgenic Arabidopsis had enhanced osmotic stress tolerance, and MYB TFs were induced by exogenous ABA to regulate stomatal movement (Yu et al., 2019; Dossa et al., 2020). In our study, the MYB family contained the largest number of TFs. MYB TFs were abundantly expressed in T2, and downregulated MYB showed higher expression, indicating that the negative regulation of MYB was more obvious. These MYB TFs may play key roles in the regulation of drought resistance. The bHLH TF family is the second largest transcription factor family in plants, among which MYC2 can activate the expression of JA-related genes (Xu et al.). In our study, MYC2 TFs were downregulated, which inhibited the expression of downstream genes, slowing down the senescence process of plants. Most of the bHLH TFs downregulated in T2 showed that bHLH TFs mainly played a negative regulatory role under drought stress. WRKY TFs also directly participate in the drought response process through phytohormone and osmotic regulation under drought stress (Maleck et al., 2000). The overexpression of loquat EjWRKY17 enhanced the drought tolerance of transgenic lines, which showed lower water loss and electrolyte leakage (Wang et al., 2021). In our study, WRKY TFs were abundantly expressed in T2, and 35 WRKY TFs were upregulated. TFs can interact with plant hormones to jointly regulate the stress resistance process of plants. In our study, the NAC and AP2-EREBP TF families were mainly upregulated during drought stress, activating the expression of downstream genes. The large upregulated expression of TFs indicated that the drought environment transmitted the stress signal to the aerial parts, which played a positive role in the regulation of the stress environment. Overall, TFs clearly play a crucial role in the responses of *H. myrtifolia* to drought stress.

## Conclusion

The morphological observation and physiological determination in our study revealed that following dehydration, leaf margins curl and become lighter, and the RWC decreased and REL increased. The transcriptome analysis of drought-stressed *H. myrtifolia* detected 62,015 unigenes and 14,272 DEGs, revealing a molecular-level drought resistance mechanism. The functional annotation of DEGs found that drought significantly affects plant hormone signal transduction, photosynthesis, glutathione metabolism, and ascorbate and aldarate metabolism, among other processes. Plant hormone signaling molecules and many transcription factors, such as MYB, NAC, bHLH, WRKY, and HSF, were activated to induce the expression of key downstream genes involved in drought response. Many photosynthesis-related genes were downregulated, and antioxidant enzyme genes were also regulated to protect the balance and maintain the stability of metabolic processes. The results of this study will fill the gap in research on abiotic stress molecules in *H. myrtifolia*; it will provide abundant genetic resources for subsequent research and breeding work. It also provides a theoretical basis for the study of the drought resistance of other ornamental plants and promotes the construction of water-saving and drought-resistant gardens.

## Data Availability Statement

The datasets presented in this study can be found in online repositories. The names of the repository/repositories and accession number(s) can be found below: https://www.ncbi.nlm.nih.gov/, PRJNA804698.

## Author Contributions

LL and JW performed the experiments. LL, JW, QW, and QM performed transcriptomic analysis. LL, MJ, SH, LS, GZ, and YZ wrote and reviewed the manuscript. CG provided the funds. All authors contributed to the article and approved the submitted version.

## Conflict of Interest

The authors declare that the research was conducted in the absence of any commercial or financial relationships that could be construed as a potential conflict of interest.

## Publisher’s Note

All claims expressed in this article are solely those of the authors and do not necessarily represent those of their affiliated organizations, or those of the publisher, the editors and the reviewers. Any product that may be evaluated in this article, or claim that may be made by its manufacturer, is not guaranteed or endorsed by the publisher.

## Footnotes

1
http://trinityrnaseq.sourceforge.net/

2
http://sourceforge.net/projects/tgicl/

3
www.r-project.org
