# Supplementary material for: Transcriptome Approach Reveals the Response Mechanism of Heimia myrtifolia (Lythraceae, Myrtales) to Drought Stress
Source: Front Plant Sci. 2022 Jul 8;13:877913. doi: 10.3389/fpls.2022.877913 (PMC9305661; doi:10.3389/fpls.2022.877913)
Supplement: Supplementary file 2 [file Data_Sheet_2.docx]

Supplementary Material

## Supplementary Figures


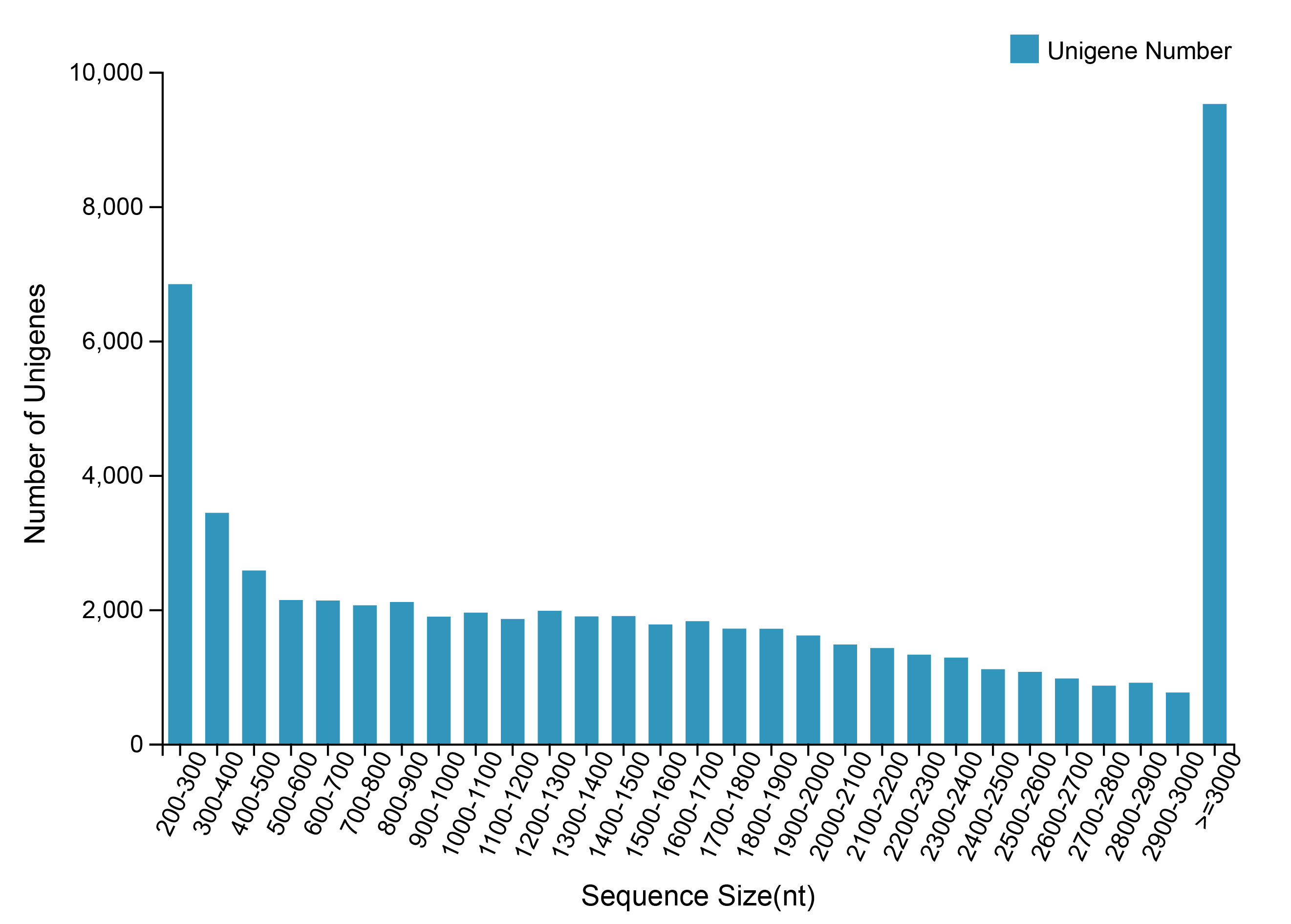


**Supplementary Figure 1.** The unigenes sequence length distribution.


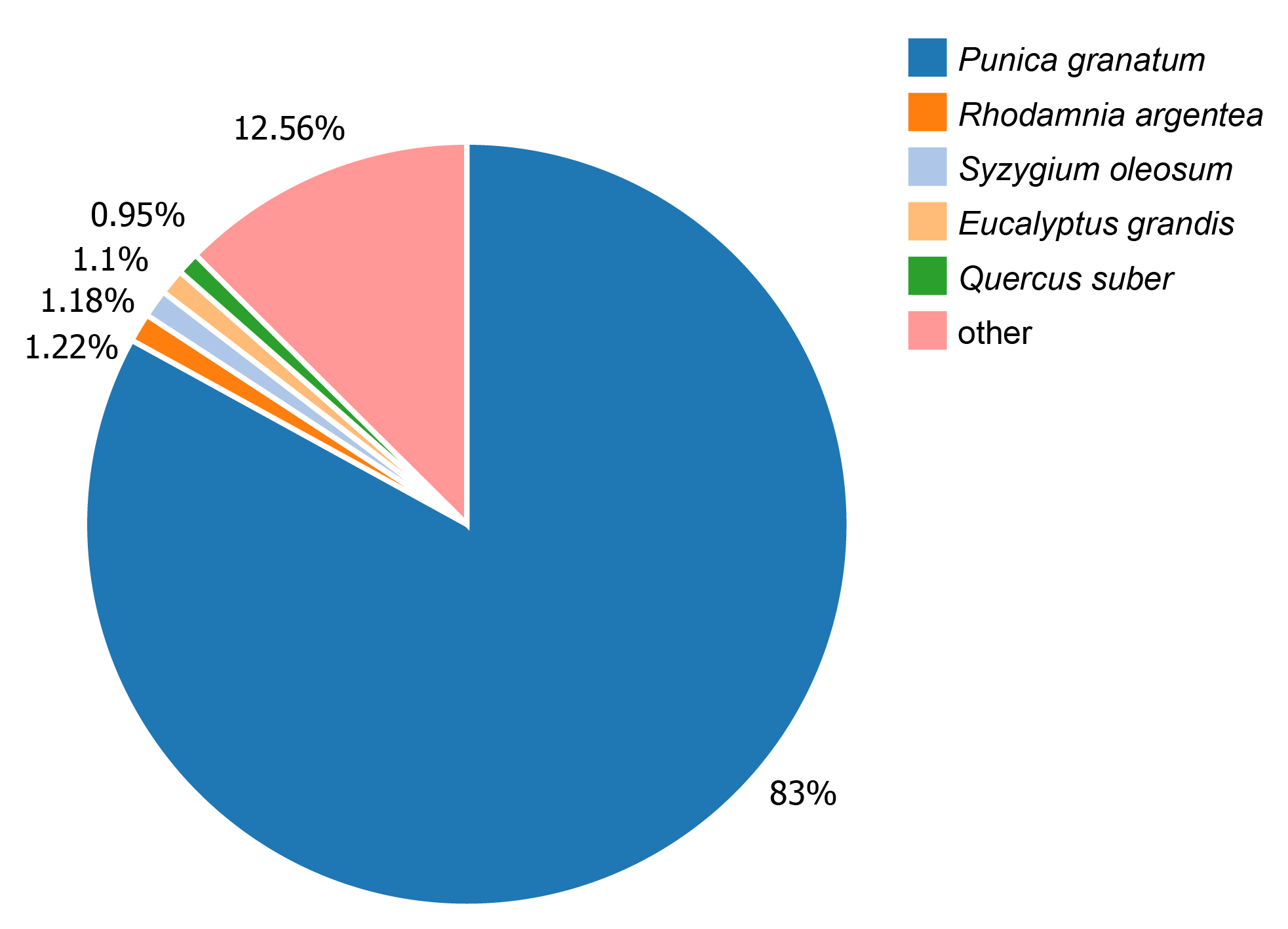


**Supplementary Figure 2.** The blast hit species based on the unigenes annotation.


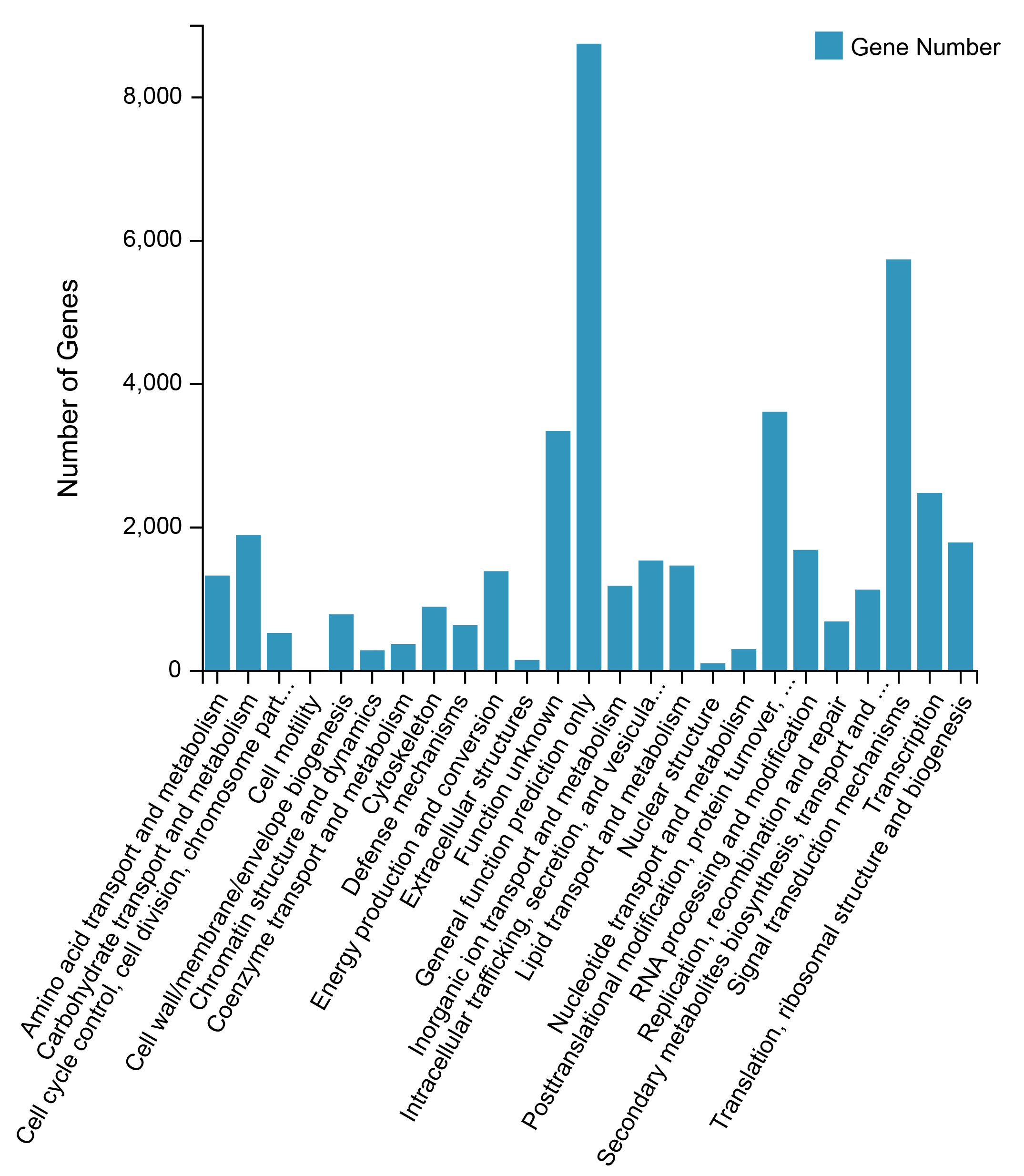


**Supplementary Figure 3.** KOG function classification of assembled unigenes.


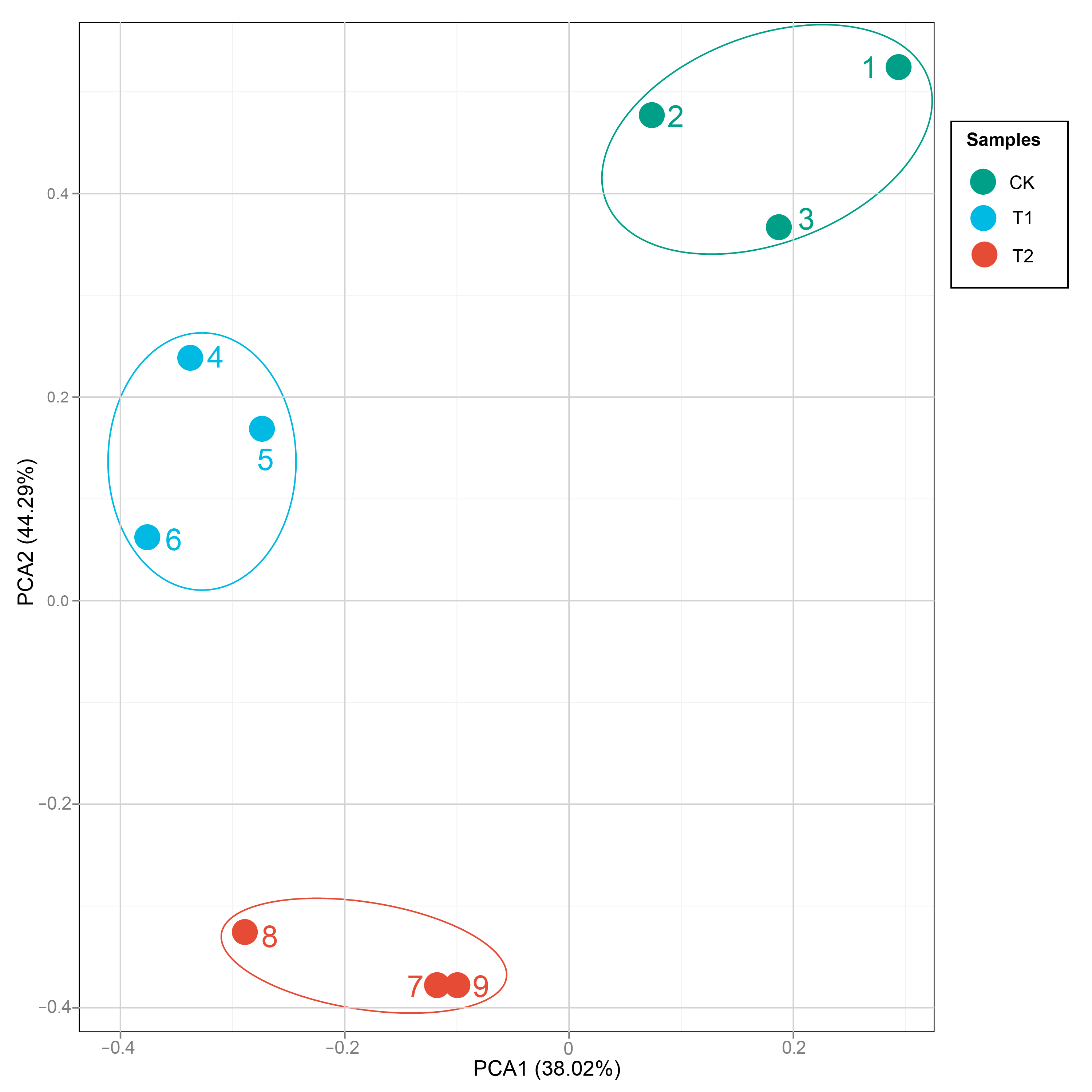


**Supplementary Figure 4.** Principal component analysis (PCA) based on the differential expressed genes (DEGs).


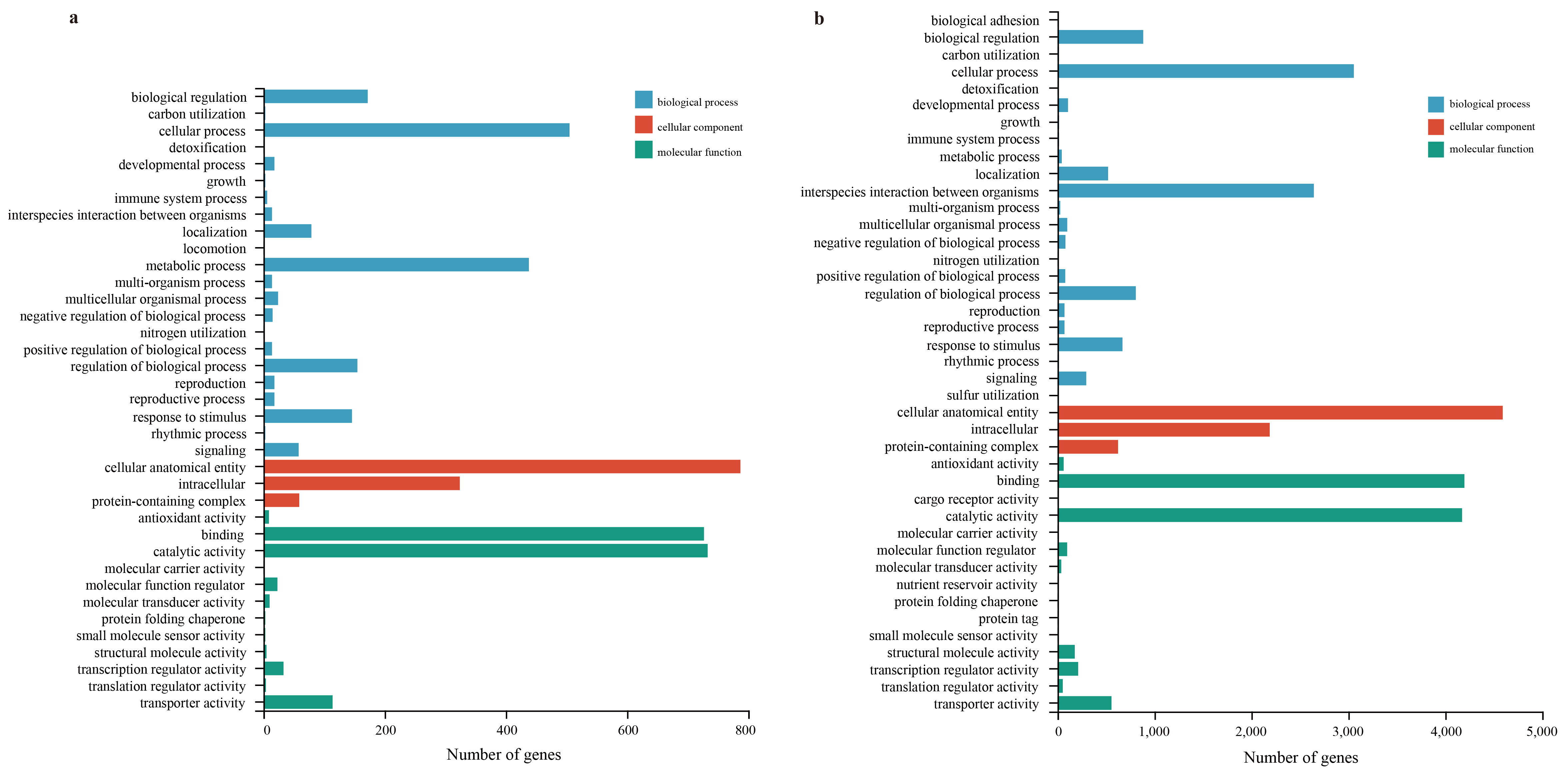


**Supplementary Figure 5.** Gene Ontology enrichment analysis of the differential expressed genes (DEGs). (a) DEGs expressed at T1 drought stress. (b) DEGs expressed at T2 drought stress.


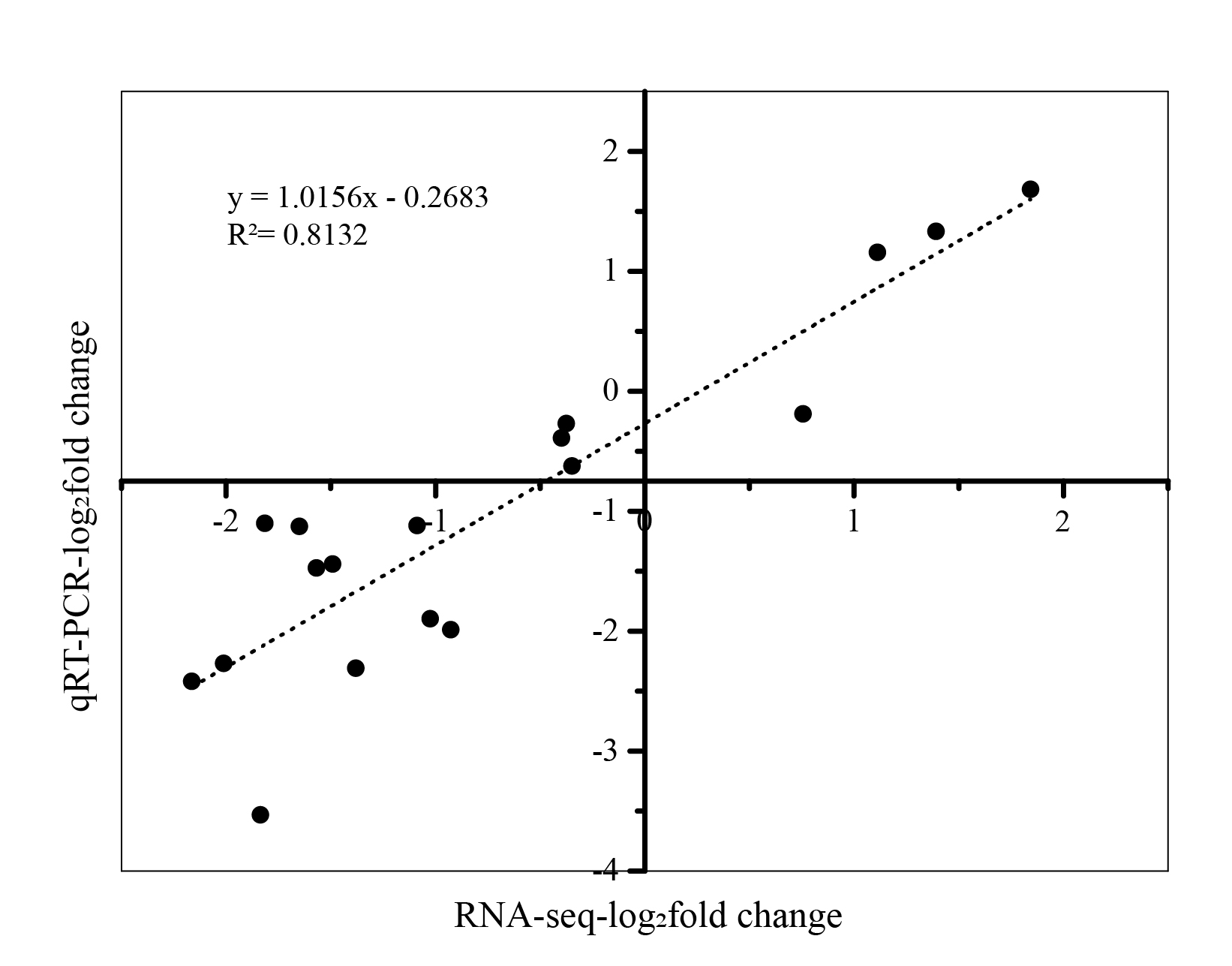


**Supplementary Figure 6.** The correlation between RNA-seq and relative gene expression.
